# INSIGHTS ON OLDER ADULTS WITH COGNITIVE IMPAIRMENT AND HEARING LOSS: END-USER AND CARE PARTNER PERSPECTIVES

**DOI:** 10.1093/geroni/igae098.1999

**Published:** 2024-12-31

**Authors:** Peter Hope, Carrie Nieman, Marcela Blinka, Paul Lee, Xueting Tu, Natalie Wang, Sevil Yasar, Esther Oh

**Affiliations:** Johns Hopkins University School of Medicine, Baltimore, Maryland, United States; Johns Hopkins University School of Medicine, Baltimore, Maryland, United States; Johns Hopkins University, Baltimore, Maryland, United States; Johns Hopkins University, Baltimore, Maryland, United States; Johns Hopkins University, Baltimore, Maryland, United States; Johns Hopkins University, Baltimore, Maryland, United States; Johns Hopkins University School of Medicine, Baltimore, Maryland, United States; Johns Hopkins University, Baltimore, Maryland, United States

## Abstract

Hearing loss is prevalent among persons aging with cognitive impairment and can have significant repercussions for individuals and their care partners. However, hearing aid usage is low among older adults, particularly among individuals with cognitive impairment. To understand the barriers and facilitators to hearing care among persons living with cognitive impairment and their care partner and to inform the development of a hearing care intervention, we conducted 20 semi-structured interviews with 10 participants with cognitive impairment and their care partners. Participants were recruited from the Johns Hopkins Memory and Alzheimer’s Treatment Center. We used heterogeneous purposive sampling to recruit participants from diverse race, ethnicities, education, and income levels. Qualitative content analysis was used to identify themes. Among end-users and care partners, 30% self-identified as African American, 45% as male, and 40% reported less than a college degree. Participants with cognitive impairment had a mean score of 16.8 (SD8.2) on the Montreal Cognitive Assessment and mean speech frequency pure tone average of 44.9 dB HL (SD12.7). End-users and care partners identified barriers to accessing care, including stigma, cost of hearing aids, lack of availability of hearing care, and participants’ denial of hearing difficulties. Suggested facilitators for improving access to hearing care include affordable hearing aids and incorporating hearing screenings into routine care. The participants’ desire to improve communication and care partner support motivate accessing hearing care. Our findings suggest that addressing barriers, promoting affordability, and enhancing accessibility to hearing care are critical in extending care to individuals aging with cognitive impairment.

